# ROS1 mutation non-small cell lung cancer—access to optimal treatment and outcomes

**DOI:** 10.3332/ecancer.2019.900

**Published:** 2019-01-29

**Authors:** Amit Joshi, Nikhil Pande, Vanita Noronha, Vijay Patil, Rajiv Kumar, Anuradha Chougule, Vaishakhi Trivedi, Amit Janu, Abhishek Mahajan, Kumar Prabhash

**Affiliations:** 1 Department of Medical Oncology, TMH, Mumbai 400012, India; 2 Department of Pathology, TMH, Mumbai 400012, India; 3 Department of Radiology, TMH, Mumbai 400012, India

**Keywords:** adenocarcinoma lung, ROS1 positive, crizotinib

## Abstract

**Introduction:**

ROS1 oncogenic fusion, which was first identified by Rikova et al, is reported to be present in 1%–2% of non-small cell lung cancers (NSCLCs) and is defined as a distinct molecular sub-group. Crizotinib is very effective in ROS1-positive patients and is now Food and Drug Administration (FDA) approved for the treatment of patients with advanced ROS1-positive NSCLC. We report our experience in a tertiary cancer care hospital in India in ROS-1 positive patients.

**Materials and method:**

The present series is a retrospective analysis of 22 patients from the prospectively maintained lung cancer audit. Demographic data were collected which included age, performance status, gender, stage, co-morbidities, sites of metastasis and smoking history. Data were also collected regarding the source of financing for crizotinib whether self-financed, through insurance or Non-Governmental Organisation (NGO) sponsored. Patients who had tested negative for epidermal growth factor receptor (EGFR) and anaplastic lymphoma kinase (ALK) and were subsequently found to be ROS1-mutation negative by fluorescence in situ hybridization (FISH) were evaluated on similar lines. All the data were entered and statistical analyses were performed using the SPSS software version 22.0. Response evaluation was done by RECIST 1.1 criteria.

**Results:**

Between January 2015 and December 2017, there were 22 patients who were ROS1 positive from a total of 535 patients in whom ROS1 testing was performed. A total of 16 patients could receive crizotinib and 6 patients were never exposed to crizotinib. Among the 16 patients who received crizotinib, 2 (12.5%) achieved complete response (CR) as their best response and continue to remain in CR at follow-up. 13 (81%) had a partial response as best response and of which on follow-up 5 (38%) have progressed, while 8 (62%) continue to maintain response. The patients who were on crizotinib had good tolerance with none experiencing any grade 3/4 toxicity. The median follow-up of the entire cohort was 15.2 months in ROS1-positive cohort and 11.4 months in ROS1-negative cohort. In ROS1-positive cohort median, progression-free survival (PFS) was not reached and the estimated 2-year PFS was 54% and in ROS1-negative cohort, it was 5.1 months. The median overall survival of the entire ROS1-positive cohort was not reached and the estimated 1- and 2-year overall survival (OS) was 72% and 54%, respectively, and was 8.8 months in ROS1-negative cohort.

**Conclusion:**

ROS1 rearrangement with an incidence of 4% of lung adenocarcinoma which is EGFR and ALK negative represents an important targetable driver mutation in the Indian population. Crizotinib also represents an effective treatment option with outcomes similar to those reported. Access to treatment remains an important roadblock to improve outcomes but innovative methods may improve access to these drugs.

## Background

ROS1 oncogenic fusion, which was first identified by Rikova et al [1], is reported to be present in 1%–2% of non-small cell lung cancers (NSCLCs) which defines a separate molecular disease sub-group [2]. The kinase domains of anaplastic lymphoma kinase (ALK) and ROS1 share a significant homology in amino acid identity and most of the differences that exist occur in conservative regions which do not have an impact on crizotinib binding and also sharing similar demographic profiles [3]. Crizotinib is very effective in ROS1-positive patients and is now Food and Drug Administration (FDA) approved for the treatment of patients with advanced ROS1-positive NSCLC [4]. Ceritinib has also been found to be effective in this group of patients [5]. However, the use of these drugs in ROS1 patients has been based on phase II studies in 50 and 32 patients, respectively; both of which had no representation from the Indian subcontinent, where the data from routine practise are sparse [5, 6]. Therefore, a retrospective analysis on a cohort treated in India is important to evaluate the treatment outcomes. We report our experience in a tertiary cancer care centre in ROS1-positive patients.

## Material and methods

### Patient selection

The present series is a retrospective analysis of a prospectively maintained lung cancer audit of patients treated from January 2015 to December 2017. The lung cancer audit is an Institutional Ethics Committee approved observational protocol and is registered with the Clinical Trials Registry India (CTRI/2013/01/003335). All patients who underwent ROS1 testing were included in this analysis.

### Pre-treatment evaluation

Demographic data were collected which included age, performance status, gender, stage, co-morbidities, sites of metastasis and smoking history. Radiological assessments were done at baseline for response evaluation in future. The ROS1 testing was performed by either of the following methods:

- Fluorescence in situ hybridization (FISH)–Formalin fixed and paraffin—embedded cell blocks were used to evaluate the ROS1 status. The FISH analysis was performed using the SPEC ROS1 dual-colour break apart probe according to manufacturer instructions.
- Next-generation sequencing–Cell block was used for ROS1 analysis for one patient.

### Treatment and follow-up

Data were collected for the type of systemic treatment patients received. This included whether the patient had received crizotinib or not, timing for starting crizotinib, break in crizotinib and the reason for stopping the treatment with crizotinib. Data were also collected regarding the source of financing for crizotinib whether self-financed, through insurance or NGO sponsored. We have a patient navigation programme wherein a social worker guides the patients for access to drugs from various resources. Data for side effects were collected as per CTCAE version 4.02. Information related to impact of treatment on the symptoms of patients and on the imaging done for response evaluation was collected. Response evaluation was done as per RECIST version 1.1 criteria. ECG was done at each routine visit. Full blood counts, biochemistry was done initially every two weeks for 2 months and then once every 2–3 months. Interval restaging computerised tomography scans were repeated every 2–3 months.

### ROS1 mutation-negative patients

Patients who had tested negative for epidermal growth factor receptor (EGFR) and ALK and who were subsequently found to be ROS1 mutation negative by FISH were evaluated on similar lines as those who were ROS1 positive. The treatment strategy for them consisted of chemotherapy, upfront tyrosine kinase inhibitors (TKI) for patients not fit to receive chemotherapy or best supportive care. These mutation-negative patients were compared for demographic details and outcomes with mutation-positive patients. In ROS1-negative patient’s demography, data were collected for all patients but the outcome data were collected for patients who visited at least once after the planning of treatment.

### Statistical analysis

All the data were entered and statistical analyses were performed using SPSS software version 22.0. Descriptive statistics were performed for the demographic data. Median follow-up was calculated for the surviving patients from the date of diagnosis to the date of the last follow-up. Patients who had not progressed at the time of last follow-up were censored. Progression was defined as clinical deterioration or radiological progression. Progression-free survival was calculated from the date of starting oral TKI to the date of progression or date of death if patients died before disease progression or date of change of treatment before progression of the disease. Overall survival was calculated from the date of diagnosis to death from any cause. Kaplan Meier curve was plotted for the progression-free survival and the overall survival in months. Log-rank test was used to compare the progression-free survival (PFS) and overall survival (OS) in different groups.

## Results

Between January 2015 and December 2017, 535 patients underwent ROS1 testing and 22 resulted positive (Table 1). The median age of the ROS1-positive cohort was 54 years (range 45–70 years) and it was 60 years (range 16–84 years) in the ROS1-negative cohort. In the ROS1-positive cohort, 13 (60%) patients were men and 9 (40%) women. Similarly, the ROS1-negative cohort had 372 (72%) males and 141(28%) females. In the ROS1-positive cohort, 3(13%) were smokers and 240 (47%) smokers in the ROS1-negative group. Co-morbidities were matched between the two cohorts with 7(31%) having hypertension and 4(18%) having diabetes mellitus in the ROS1-positive group and 123(24%) and 95 (18%), respectively, in the ROS1-negative group.

In the ROS1-positive cohort, 21(95%) patients had adenocarcinoma histology, while 1(5%) patient had sarcomatoid adenocarcinoma as primary histology. One patient had stage III unresectable disease in the ROS1-positive cohort and 22 (4%) in the ROS1-negative cohort. The median number of sites of metastasis was 2 in both ROS1-positive and negative cohort. Opposite lung was the most common site of metastasis in both the ROS1-positive and negative cohort with 11(50%) in the ROS1-positive cohort and 265 (52%) in the ROS1-negative cohort. Pleural effusion was the next most common presentation with 9 (40%) and 231(45%) in the ROS1-positive and negative cohorts, respectively. The other common sites of metastasis were skeletal, liver and adrenals (Table 1). Brain metastases were seen in 2 (9%) patients in the ROS1-positive cohort and 71 (14%) in the ROS1-negative cohort.

### First line treatment regimens

Overall, 16 (73%) of patients were exposed to crizotinib at some point while 6 (28%) never received crizotinib. The reason why crizotinib could not be offered at any time were death before the ROS1 report became available in 2 (33%) and lack of financial feasibility in 4(67%) patients. Out of the 16 patients who received crizotinib only 2 could receive it as first-line therapy.

The ROS1-negative patient cohort comprised of 513 patients who were included for demographic analysis and 280 patients were included in outcome analysis. In the ROS1-negative cohort patients of the 513 patients, 306 (60%) received chemotherapy as first-line therapy, 164 (32%) received oral TKI as first-line therapy and 43 (8%) were planned upfront for supportive care only.

### Efficacy of crizotinib

Among the 16 patients who received crizotinib, 2 (12.5%) achieved complete response (CR) as their best response and continue to remain in CR at follow-up. 13 (81%) had a partial response as best response and of which on follow-up 5 (38%) have progressed while 8(62%) continue to maintain response. There was only one patient who progressed on the first assessment. Of the five patients who had progressed on therapy, one patient received Ceritinib, two were offered chemotherapy, one patient died on progression and another one was continued on crizotinib beyond progression as the progression was asymptomatic and the patient was not willing to receive any second-line therapy. The patient on ceritinib had stable disease as the best response and subsequently at progression after a period of 6 months best supportive care was offered due to poor performance status. In those who were offered chemotherapy, one patient had stable disease and in the second patient response to treatment was not available.

### Survival outcomes

**Figure 1.**
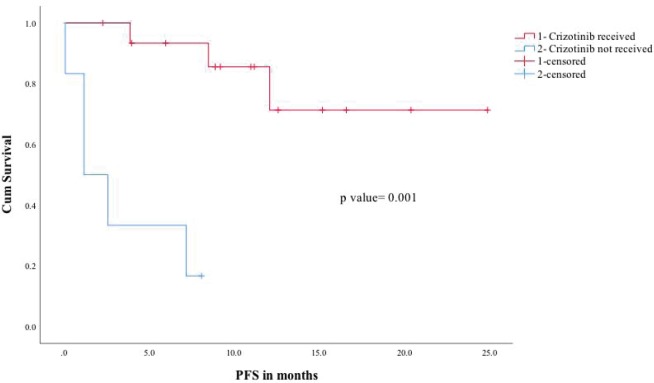
PFS for crizotinib received (1) versus crizotinib not received (2).

**Figure 2.**
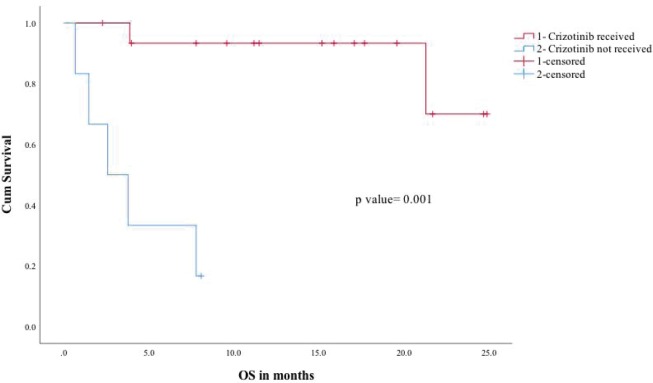
OS for crizotinib received (1) versus crizotinib not received (2).

The median follow-up of the entire cohort was 15.2 months in ROS1-positive cohort. In ROS1-positive cohort median, PFS was not reached and the estimated 2 year PFS was 54%. In a univariate analysis for PFS, factors included were age (under or over 60), gender, performance status (Eastern Cooperative Oncology Group (ECOG) 0–1 versus 2 or more), crizotinib usage and brain metastases (present or absent at baseline). Among all the factors, ECOG performance status 0–1 and crizotinib use predicted significantly better PFS (Figure 1). The median overall survival of the entire ROS1-positive cohort was not reached and the estimated 1- and 2-year OS was 72% and 54%, respectively (Figure 2). A similar univariate analysis was done for OS as in PFS and the same factors were compared for their impact on OS. In OS also among all factors assessed, the use of ECOG performance status 0–1 and crizotinib predicted for significant improvement in overall survival.

**Figure 3.**
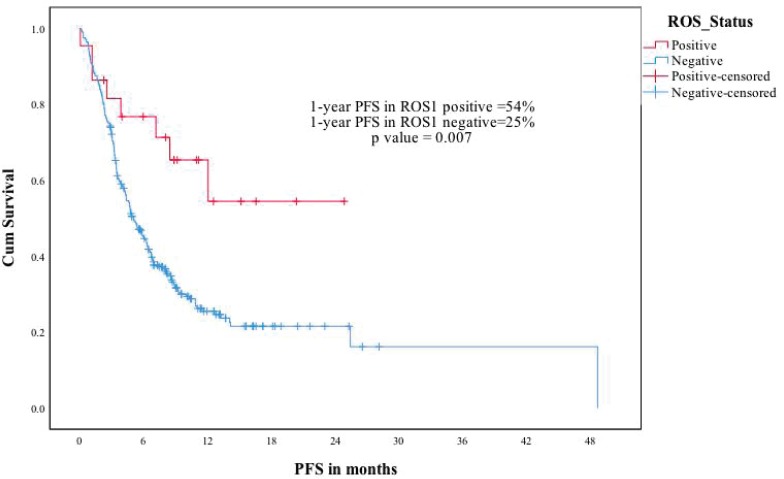
ROS1 positive versus ROS1 negative patients—PFS.

**Figure 4.**
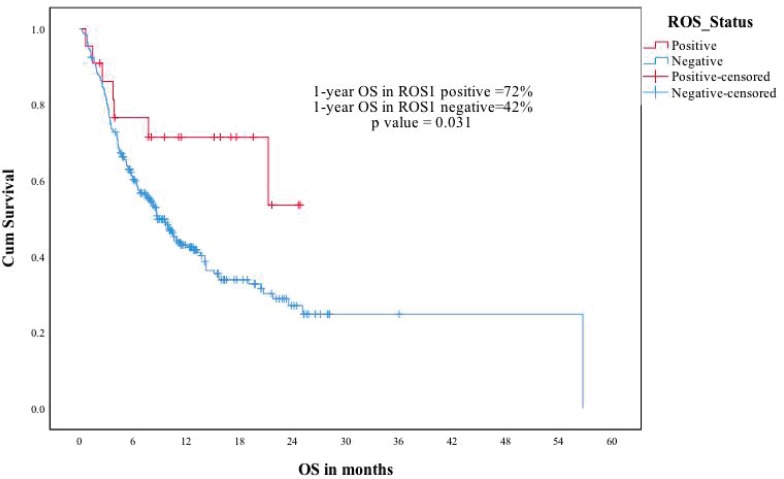
ROS1 positive versus ROS1 negative patients—OS.

The median follow-up of the ROS1-negative cohort was 11.4 months. The median PFS was 5.1 months for the 280 patients who at least visited once after treatment was planned. The median OS was 8.8 months in the ROS1-negative cohort. The ROS1-positive and negative cohorts were compared for PFS and OS. The estimated 1-year PFS was 54% versus 25%, respectively (p-0.007) and the estimated 1-year OS was 72% versus 42% (p-0.031) (Figures 3 and 4).

## Discussion

Targeted therapy and precision medicine have ushered in a plethora of new molecules which have been very impressive in trial settings but their reproducibility in the real-world population remains unknown. A number of factors such as difficulty in monitoring, heterogenous populations and accessibility to the expensive molecule remain an area of concern. It is in this scenario that we planned to examine the access of this treatment and the outcomes of these patients. We also examined tolerance of crizotinibin ROS1-positive patients from the Indian subcontinent. At the same time, we compared outcomes in ROS1-positive patients and patients who were found to be ROS1 negative.

The present analysis is the largest series of ROS1-positive patients from the Indian subcontinent. A total of 535 patient biopsies were analysed and 22 patients were found to be ROS1 positive. This 4% positivity appears to be higher as compared to previously reported incidences of 1%–2 % [2, 7]. This high positivity of ROS1-positive patients in the study population could be due to the performance of ROS1 FISH in a selected cohort of EGFR mutation and in ALK rearrangement negative patients.

In the seminal work done on ROS1-positive patients by Shaw *et al* [6] (PROFILE 1001), the median age of the study population was 55 years which was similar to our study median of 53 years. However, the study had 57% female population as opposed to ours which had only 40% females. The ROS1-negative cohort, however, had only 28% females. In the expansion cohort of their study, they have mentioned that all their patients were never or former smokers, but in our study population, there were 13% smokers [8]. However, the ROS1-negative cohort had 47% smokers. Both ROS1-positive and negative cohorts were similar in other demographics such as co-morbidities status, average number of metastatic sites and presence or absence of brain metastasis at baseline. Patients who had brain metastasis at baseline were not included in the PROFILE 1001 study but asymptomatic brain metastasis was included in the OxOnc study [6, 9].

PROFILE 1001 had an objective response rate (ORR) of 72% and was the same in the OxOnc study and the ORR in our study population was also high (93%). The ROS1 positive patients had better OS and PFS as compared to ROS1-negative patients, especially when ROS1-positive patients who received crizotinib were compared with ROS1-negative patients. ROS1-positive patients who could not receive crizotinib were small in number but their outcome was dismal. This suggests that crizotinib treatment in ROS1-positive patients made the difference in outcome. In a limited resource setting like India where the cost of crizotinib is 82,000 rupees per month (approx. USD-1200) and where a significant majority are uninsured this is a major obstacle. We trained and appointed a patient navigator who helped ROS1-positive patients in securing crizotinib. Patient navigation has been proven to be a useful intervention in addressing social disparities in many cancers and in lung cancer patients for treatment initiation and adherence [10, 11]. The patient navigator acted as a single point of contact between patients and various NGO’s and helped patients with paperwork and ensured drug procurement assistance at follow-up. This ensured that approximately 30% of patients received crizotinib totally free of cost who would otherwise be deprived of the same. We have used this approach using patient navigators earlier with ALK positive patients with reasonable success. This highlights the limitation of access of effective but expensive drugs in a developing country like India but also provides a reasonably effective solution.

Crizotinib was generally well tolerated in our study population and there were no grade 3/4 adverse events (AE) (Table 2). The most common side effect was elevated transaminases which was seen in 19% and was similar to that of the PROFILE 1001 (22%) study but appeared better than 55% seen with the OxOnc study. This difference could in part be a reflection of the different ethnic populations. The percentage of other toxicities recorded in our study population also appears to be significantly lesser than that of these two studies. These differences may be because of the retrospective nature of our work and both the other studies were done in trial settings with very rigorous recordings. However, there was toxicity related drug interruption in one patient and we could start subsequently with no dose adjustments.

## Conclusion

ROS1 rearrangement with an incidence of 4% of lung adenocarcinoma which are EGFR and ALK negative represents an important targetable driver mutation in the Indian population. Crizotinib also represents an effective treatment option with outcomes similar to those reported. Access to treatment remains an important roadblock to improve outcomes but innovative methods may improve access to these drugs.

## Funding statement

This research did not receive any specific grant from funding agencies in the public, commercial, or not-for-profit sectors

## Conflicts of interest

The authors have no conflict of interest to declare.

## Figures and Tables

**Table 1.** Demographic data and baseline tumour characteristics.

	ROS1 positive number (%)	ROS1 negative number (%)
Number of patients	22	513
Median age	54	60
Gender		
• Male	13 (60%)	372 (72%)
• Female	09 (40%)	141 (28%)
Smoking history	03 (13%)	240 (47%)
Co-morbidities		
• Hypertension	07 (31%)	123 (24%)
• Diabetes Mellitus	04 (18%)	95 (18%)
Stage of disease		
• Stage III	1 (5%)	22 (4%)
• Stage IV	21 (95%)	491 (96%)
Median number of metastatic sites	2	2
• Opposite lung	11 (50%)	265 (52%)
• Brain metastasis	2 (9%)	71 (14%)
• Pleural effusion	9 (40%)	231 (45%)
• Liver	2 (9%)	70 (14%)
• Adrenals	6 (27%)	51 (10%)
• Bones	8 (36%)	154 (30%)
Procurement of crizotinib *n* = 16		
Self, no insurance	7 (44%)	
Self, insurance	4 (25%)	
Sponsored by NGO	5 (31%)	

**Table 2.** Safety and AE with crizotinib.

	Number (%)
Adverse event	
• Visual Hallucination	01 (6.2%)
• Anaemia	02 (12.5%)
• Aspartate aminotransferase (AST)/alanine aminotransferase (ALT)	03 (18.7%)
• Neutropenia	01 (6.2%)
• Diarrhoea	00
• QTc prolongation	02 (12.5%)
• Bradycardia	01 (6.2%)
• Renal insufficiency	02 (12.5%)
Drug interruptions	
• Toxicity	01 (6.2%)
• Lack of compliance	02 (12.5%)
• Drug unrelated	00
Dose reductions	00
